# A qualitative study exploring parents’ experiences with epinephrine use for their child’s anaphylactic reaction

**DOI:** 10.1186/s13601-018-0230-y

**Published:** 2018-10-18

**Authors:** Rishma Chooniedass, Beverley Temple, Donna Martin, Allan Becker

**Affiliations:** 1 John Buhler Research Centre, College of Medicine, Faculty of Health Sciences, University of Manitoba, 504H 715 McDermot Ave, Winnipeg, MB R3E 3P4 Canada; 2 Helen Glass Centre for Nursing, College of Nursing, Faculty of Health Sciences, University of Manitoba, 89 Curry Place, Winnipeg, MB R3T 2N2 Canada

**Keywords:** Anaphylaxis, Epinephrine, Food allergy, Barriers, Education, Management

## Abstract

**Background:**

Children with life threatening food allergies live with the constant threat of a fatal reaction, and caregivers must always be prepared to treat with an epinephrine auto-injector (EAI). This interpretive phenomenological study explored parents’ perceptions and lived experiences with prescribed EAI use for their child.

**Methods:**

The purposive sample included ten parents of five children under 12 years of age, diagnosed with a food allergy and prescribed with an EAI who recently experienced anaphylaxis. Data sources included digitally-recorded semi-structured interviews and a reflexive journal.

**Results:**

Eight main themes emerged: perception of anaphylaxis, life challenges, isolation, anxiety, hesitation, guilt, influence of health care professionals, and lessons learned. Parents uniformly described multiple life challenges and feelings of isolation, anxiety and hesitation during a reaction that lead to subsequent guilt.

**Conclusions:**

Handling reactions correctly provided parents with confidence to treat subsequent reactions. Witnessing the effects of an EAI and receiving positive feedback from health care providers further strengthened their confidence to quickly and competently intervene in future reactions.

## Background

Food allergy has increased two to threefold in the last 20 years and is associated with increased anxiety, poor quality of life and increased health care costs including emergency visits and hospital admissions [1, 2]. Approximately 8% of children in North America have a self-reported food allergy [3]. It is estimated that food allergies cost the American economy approximately 25 billion dollars (USD) annually with direct and indirect costs of medical appointments, including loss of family work days [4] and the cost is only increasing as incidence of food allergy rise [1]. This medical condition is of global concern affecting quality of life of both patients and their families.

Presently, there is no cure for food allergies. Avoidance is the primary strategy with epinephrine as the medical treatment recommended for an anaphylactic reaction [5]. Anaphylaxis is a severe allergic reaction that can affect multiple organ systems in the body and can rapidly cause death. Rates of epinephrine use during an anaphylactic reaction are alarmingly low and treatment is often inappropriately managed [6], while treatment in children is often withheld. The reasons for this range from individuals not carrying an epinephrine auto-injector (EAI) when a reaction is occurring, to caregivers not wanting to administer medication if they are unsure of themselves or the situation. Some speculate that injecting epinephrine into their child is a traumatic event which may contribute to the profound lack of treatment [7]. It is crucial to understand the root cause(s) of failing to treat anaphylactic reactions competently in order to better manage the health of children with food allergies.

Unsworth states, “comprehensive and helpful published advice on patient training (for EAI) is available, but compliance remains poor” [8]. If health care providers are to improve outcomes of anaphylactic reactions, then researchers must identify why caregivers delay or neglect to use EAIs. There is a lack of research exploring experiences and attitudes related to managing food allergies and the use of EAIs [9]. By understanding the thoughts and emotions of parents who have witnessed their child’s anaphylactic reaction, health care providers can ensure that caregivers develop the skills, education, and support necessary to make certain that the next reaction is appropriately treated. The purpose of this qualitative study was to describe parents’ perceptions and experiences with epinephrine use during a child’s anaphylactic reaction.

## Methods

This project used steps outlined by Creswell to perform data analysis and interpretation of findings from one-on-one interviews with parents of children living with a life threatening food allergy [10]. Using a purposive sample, a semi-structured, open ended, face-to-face, one-on-one, in person interview was conducted with each participant.

Approvals by the Research Ethics Board (E2015:039) and Health Research Impact Committee (RI2015) were obtained prior to starting the study. Following obtaining consent, demographic data were collected to describe the sample population. Each participant was asked to tell the story of their child’s anaphylactic reaction, outlining their experiences, thoughts and feelings during that critical event.

### Inclusion criteria

Inclusion criteria were parents and caregivers of children under age 12 with a previous diagnosis of food allergy confirmed by a Pediatric Allergist, who had experienced an anaphylactic reaction that required an emergency room visit within the last 24 months. They must have previously filled a prescription for an EAI. The caregivers were required to speak English.

### Exclusion criteria

Exclusion criteria included any caregivers who did not speak English, had an anaphylactic reaction more than 24 months ago, or did not have an EAI. Also, caregivers of children living with another major chronic illness were excluded as this may further negatively affect their perception of health or reasons for use of EAI.

### Sampling and recruitment

Participants were recruited by a nurse educator from The Children’s Allergy & Asthma Education Centre or via posters displayed at the Children’s Allergy Clinic.

Ten participants were recruited. Interviews ranging from 30 to 80 min were conducted in participant homes or workplaces; and recruitment ceased after the tenth interview as no new themes emerged. Data sources included transcripts, field notes, and memos. Audio recordings were collected and transcribed verbatim by an independent transcriptionist. As a form of verification, random copies of transcripts were reviewed by another researcher to ensure that there was congruence in theme development. All data sources were imported into NVivo v11.2 software (2010).

## Results

### Demographics

Ten parents participated (five females) aged 32–46 years (Table 1). Five children were described in this study who were 3–10 years of age and parents self-reported their child’s multiple food allergies (see Table 2). Four of the children were diagnosed within the first year of life, and one was diagnosed at age three. It was not confirmed whether each allergen sensitization caused clinical symptoms as only the most severe anaphylactic reaction within the last 24 months was described.

**Table 1:** Study participant demographics

Variable	Frequency	
	N	Percent
**Caregiver**		
Mother	5	50
Father	5	50
**Employment status**		
Employed	8	80
Self-employed	1	10
Unemployed	1	10
**Education**		
High school	2	20
Post secondary degree	8	80
**Household income**		
$75,000–$100,000	2	20
$100,000–$125,000	2	20
$125,000–$150,000	2	20
> $150,000	4	40

**Table 2:** Child demographics

	μ ± SD
Child age	6.3 ± 2.9
Number of allergies	4.6 ± 2.1
Number of anaphylactic episodes	4.9 ± 2.1
EAI uses	3.2 ± 1.9

Variable	Frequency	
	N	Percent
**Age at diagnosis**		
≤ 1 year	4	80
1–5 years	1	20
**Parent recollection of initial diagnosis**		
Allergist	6	60
Emergency doctor	2	20
Nurse practitioner	1	10
Can’t recall	1	10

The length of time participants lived with their child’s food allergy ranged from 2 to 9 years. Every child had an EAI and kept it present at all times. All children had experienced an anaphylactic reaction at least two and some up to nine reaction requiring an Emergency Department visit within 24 months. Epinephrine administration from participants ranged from one to six times. As a form of cross verification, data triangulation from hospital charts was obtained to validate participant’s recall.

**Fig. 1:**
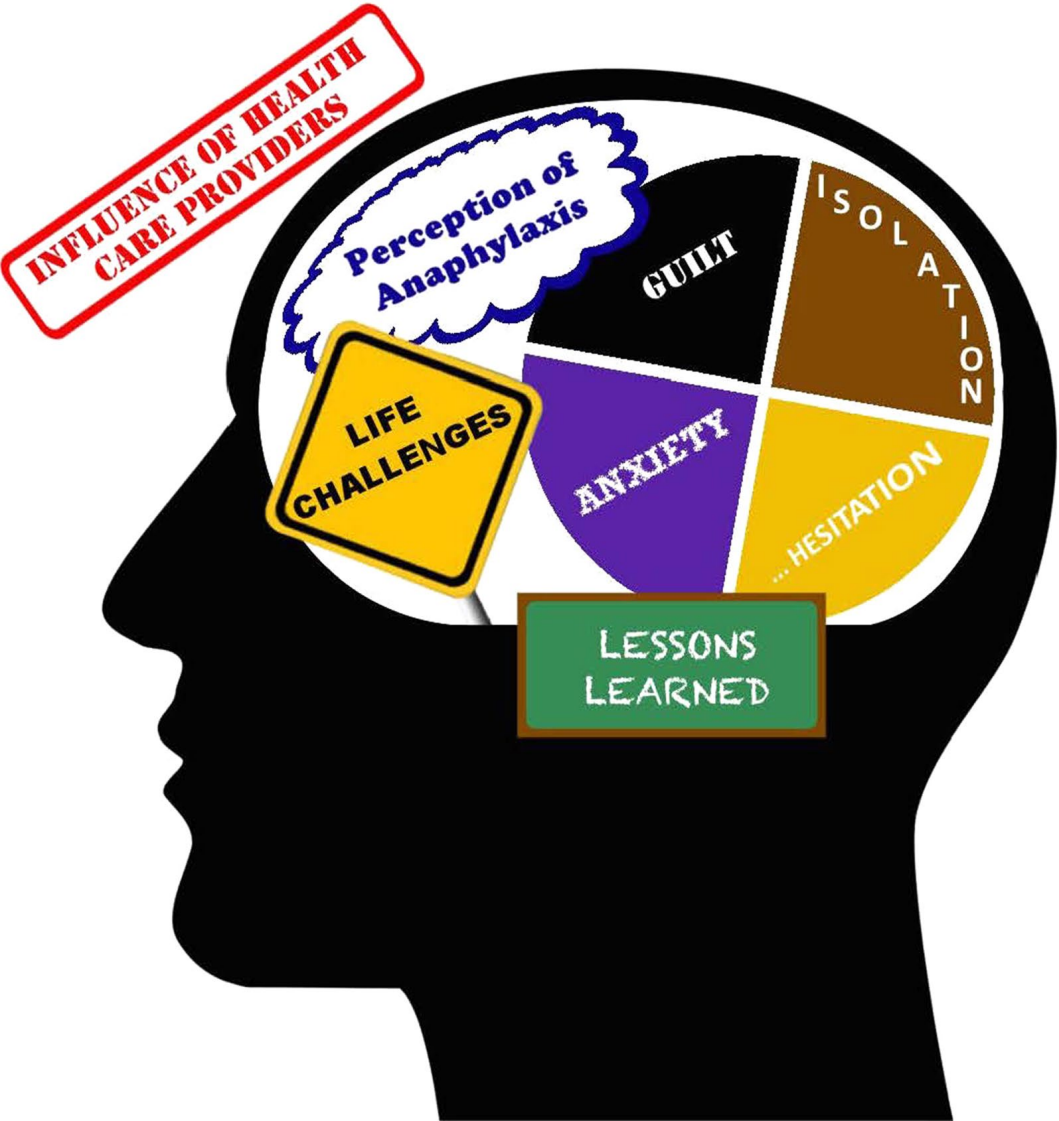
Emerging themes

Analysis uncovered a range of thoughts and emotions experienced by parents when their child suffered an anaphylactic reaction that could impact their decision to use an EAI. Figure 1 illustrates the eight main themes that emerged from this study: perception of anaphylaxis, life challenges, isolation, anxiety, hesitation, guilt, influence of health care professionals and lessons learned. This figure represents the external and internal influences on a caregiver’s perception. The following section will present the major themes that emerged from the data.

### Perception of anaphylaxis

Participants were asked: ‘What does the term anaphylaxis mean to you?’ They constructed their own meanings based on experiences or what they have been told about anaphylaxis by others.

I think we do a great disservice to people with EpiPen videos that show people going into respiratory distress and having red flushed hive-covered faces and dropping to the ground; … it doesn’t always appear that way. (F2)

This comment serves as a reminder that each reaction is unique, manifesting differently in the same person, even when triggered by the same allergen.

things are going to go into hyper overdrive and eventually shut down… You’re in a race for time to make sure that things get fixed properly in a timely fashion. (M2)

The perception that the body’s systems “shut down” and using an analogy of a race, exemplifies how rapidly the reaction progresses. It describes how fast anaphylaxis occurs and how quickly caregivers must respond, without relying on specific detailed symptoms.

### Life challenges: “logistically expensive nightmare”

Many participants explained how every aspect of their life changed. The obvious and immediate change was avoidance of the suspect allergen. Some participants explained the challenges associated with avoiding allergens. For instance, a caregiver of a child with dairy allergy expressed the additional burden of sourcing foods that do not contain milk and eggs. In addition, it was noted that if the child had multiple food allergies it made avoidance and shopping much more complicated.

Society integrates food into social activities, including family gatherings, meals at restaurants, school, team activities, or celebrations. The task of constantly checking, and re-checking food labels while grocery shopping became an added burden that families had to face on a daily basis. Sourcing alternative foods is possible but requires both more work and necessitates more expense.

Dealing with the high cost of these foods is intense, and we are both middle-income earners. Good grief, what do low income people with allergies do? (F2)

At 110 bucks it’s like whoa eh, hold on. You know, never mind the ambulance rides… people can’t afford that epinephrine. It’s expensive. (M1)

Not only is it a challenge to vigilantly shop for foods that are allergy safe for their children, but the expense is felt in the cost of food, medication, and ambulance fees. Even patients who have stable incomes can feel the burden of paying for an auto injector, further compounded when they are often advised that they should have one for home, school, backpack and other locations. Some participants went as far as discussing that travelling is a major challenge that is best avoided. Preparations for travel included filling multiple prescriptions of EAIs, finding accommodations with a kitchen to prepare meals, and plotting out nearby grocery stores and hospitals.

### Isolation, limited social support, misunderstood

Another significant challenge was a change in relationships within the extended family, friends and other support groups. These challenges had the greatest impact on the quality of life experienced by those living with a food allergy and their families.

(Anaphylaxis is) basically life or death, it’s serious and that you don’t take chances. And that people don’t understand it unless you’ve seen your child basically starting to die in front of you don’t, they don’t get it. And even now, some of our family doesn’t quite get it. (F3)

The frustration becomes evident when one’s own family does not understand the implications of anaphylaxis. Another participant expressed frustration with his family because they continued to put his child at risk of a reaction and he believed that the best action was to avoid visiting their home. The safety of this participant’s child is the primary concern, regardless if this meant isolating from family members.

It can be lonely. Um, you really find out who your friends are (crying). So you know friends that I had totally been there for just basically vanished the second that she was diagnosed. So because nobody wants their lives to be inconvenienced and food is such a big social aspect. So, things like getting together for play dates or you know all the stuff we used to do before, nobody really wants to do that. (F5)

### Constant anxiety and a visceral fear

Anxiety and fear are parental instincts that intensify when their child has been diagnosed with a food allergy. Participants described a constant feeling of anxiety, both when they are away from their child and even when they are by their side. Some expressed that anxiety may improve with time, but may also stay for years.

We’re still scared (crying)… Um, we question as parents whether he’s going to get to be able to do all the things that we want him to be able to do, because of our own fears. (F4)

It’s still a very visceral fear that’s always brooding in the back of your mind. What’s going on at school?… you’re always in a constant state of, alertness to it…Cause I can’t be there 24/7… I’ll say fear. I’m not going to lie you know. It’s this nagging background fear that something’s going to happen. (M2)

Parents live with the ever-present possibility of a severe reaction, and the morbidity associated with living in a state of constant uncertainty and anxiety.

(crying) I’m sorry but she was basically dying in the back of the car and I was sitting there in the back with her trying to like wake her up because she was starting to pass out now. (F3)

The traumatic memory relived by this participant is a stark reminder of the seriousness of this situation and reminds her of the mortality of her daughter. This statement evokes the fear and sense of helplessness that parents experience when faced with their child’s reaction. Reactions are unexpected, and not being able to control a situation can result in an overwhelming feeling of dread.

It was my fear of hurting my child even though I knew the needle was the right thing. Um, I guess, I think there was even fear of like doing it wrong. (F4)

The fear of what is perceived as inflicting more pain on one’s child is a common theme that participants described. Even when parents are witnessing their child suffering a severe reaction, the thought of giving a needle often seems an overly invasive intervention, despite the fact that they recognize it is necessary. This hesitation may cause more harm and is rationalized by the desire not to inflict pain.

### Hesitation

Hesitation was a common reaction when uncertainty made participants doubt themselves. Often it was confusion about how the child came in contact with the suspect allergen rather than issues regarding acknowledgment of the reaction. Denial, being reluctant to treat and the urge to wait to see what happens were common responses to an anaphylactic reaction.

Doubt. Self-doubt; do I, am I doing the right thing? Am I over-reacting? Am I under-reacting?… My brain didn’t say get me the EpiPen, then I’ll take him… I’ve been trained, I’ve trained people…as a public health nurse in the schools…but in the moment with the gradual onset that’s not this big, exciting, obvious thing…But I was still thinking really? Really? This is warranted?… I was questioning myself while I’m preparing to give it. (F2)

Even in well trained individuals who have experience with anaphylaxis, there is still a hesitation to act, and a sense of down-playing the severity of a situation that can quickly escalate. The inner conflict of self-doubt leading to hesitation is a commonly identified theme among participants. This parent’s response to the uncertainty of acting, and how others may view their actions serve to illustrate that caregivers are dealing with more than the critical situation of anaphylaxis. They are dealing with thoughts and fears about how to treat, when to treat, consequences of treating (hurting their child further) and what others will think of their actions. One participant described an EAI as a “horse tranquilizer” because of the size of the device. He described the anxiety associated with using something that large on a small child.

We should have given the EpiPen, but it was the fear of “I’m going to make this worse for my child.” Like he’s already dealing with the struggling… we just need to get him to the hospital because I don’t want to hurt him more. (F4)

Even when they know that a reaction is occurring, there continues to be a reluctance to use epinephrine. Once again, it is evident that hesitation during a stressful moment leads to delay in administration of epinephrine, even when the caregiver is well aware of the correct course of action, partially due to situational stressors or a “kaleidoscope of stressors”.

### Guilt

A sense of guilt stemmed from many sources. One reason for experiencing guilt was directly due to not treating the reaction. Participants expressed other reasons for feeling guilt, and often identified it was due to the way they had decided to live life and care for their child with a food allergy.

The words we use are probably pretty offensive and probably my kids should go for counselling because he probably feels like some, some kind of leper because we’re always talking about contamination and cross-contamination. (F2)

The implications of using terms like contamination (often associated with connotations such as harmful, unclean) make this parent think that her child will feel different from other children.

I should have given it. But then I still didn’t give it cause I’m almost there (hospital). And I’m just like oh crap this is serious! I’ve worked emerge (in the Emergency Department), like I just felt such shame and I felt so stupid. And like me fluffing it off, or you know watch and, watchful waiting, or, you know, you know observation is going to kill him one day and I’m trying to snap out of that. (F4)

Anaphylaxis is one illness in which ‘watchful waiting’ is a potentially life-threatening decision, and this parent’s resultant guilt stems at least in part from following such a course of action.

I think I was seriously thinking that like I could lose my son! He could die! Like, this isn’t right… I still had the EpiPen. It was still around him. It was on, on his belt. (F4)

Guilt revolves around the delayed use of the EAI for fear of having to give a needle. This guilt is magnified by how quickly her child improved upon treatment with epinephrine, and further reflects the guilt of what the parent perceives as unnecessary suffering, which could have been avoided if only they had acted sooner. The realization that there is a potential for a child’s death is something that cannot be put into words, and, at times, is inconceivable.

### Influence of health care professionals

Caregivers are reliant on health care providers for acute and long-term treatment. We trust them because of their profession and rely on them to be proficient and informed on current evidence and best practices. But how do their actions or their reactions affect us?

I know from that first time when we got to the hospital and they were like why didn’t you give the EpiPen? And they were basically like yelling at us. (F4)

This participant describes the response from emergency staff and relates feeling berated due to her lack of EAI use. This situation illustrates that parents perceive health care professionals as a source of authority. The manner in which health care providers react to patients and their families can provide a lasting impression, both positive and negative.

But then when they (doctors) don’t give it either, you know you can say okay because they’re in the hospital they can always intubate and start IVs and do all these wonderful things. But hello? Like if you really want to reinforce proper parental management in the community, you got to role model it in the hospital too. (F2)

When a participant encounters a situation when health care providers do not provide the standard of care for an anaphylactic reaction, it raises doubts in their mind about the competency of the health care provider. The action of a health care professional not administering an EAI undermines the teaching that caregivers have received regarding prompt epinephrine use.

So I called her paediatrician and I said, look this is what happening. She started to wheeze and um, he said well just put the phone up to her, up to her mouth so I can kind of hear her breathe… And he says… it likely was a reaction but basically wait until she was having trouble breathing or if she was turning blue before I called 911. (F5)

Retrospectively this parent realizes the information given was not consistent with the standard of care for an anaphylactic reaction. This results in mistrust of health care professionals and can become a barrier to future care.

We had a reaction in Alberta and the hospital is completely different than here (Winnipeg). I mean they were like, well she can still breathe so why are you even here? …She’s got four inch hives on her and she’s coughing all the time… with a diagnosis of anaphylaxis you would treat this as anaphylaxis. But they were nah it’s fine; go away. (M5)

This is another example of a patient who encountered inaccurate advice and contrary to what they have been taught. There are health care providers who take initiative to educate caregivers on proper management and treatment of anaphylaxis, and it is disconcerting to encounter other providers who disseminate incorrect information and treatment.

### Lessons learned

The best advice might be from someone who has experienced anaphylaxis. Real world advice has both the weight of personal experience and may be more directly relatable to an individual in the same circumstance. This type of education may prove more effective than artificially constructed or simulated examples from health care providers.

I have documented most of [her] reactions; I put together a book for her daycare when we had to put her into day care to say this is what all these things look like on the different sites of her body…they told us that they learned more from us than they did URIS (Unified Referral and Intake System in Manitoba that provides health care support for children in community programs). Because they, they hear okay well hives but nobody really knows what hives look like. So you know having, having a resource like that I think would have been really helpful for me to also have somebody tell me what it would be like to; the importance of documenting everything. (F5)

This family copes with lifestyle changes by becoming proactive and advocating for their child. By providing detailed information on their child’s health and possible signs of reaction, they empower themselves and, thus, are better able to deal with the challenges they must face on a daily basis.

Always think anaphylaxis even if there’s been a long delay between a potential exposure, even if you haven’t seen it. Because if you don’t see it, your tendency is to figure out what’s wrong and I’ve been caught in that trap so many times where you’re trying to figure out was there an exposure? …Rather than just recognize the symptoms and respond appropriately. (F2)

The lessons learned from this parent’s experience has allowed them to reflect upon and evaluate their initial response and coping skills. Whereas it may be second nature to try to answer the question ‘why is my child having a reaction now’, experience has taught this participant that the ‘why’ really is not the important question at the time. Rather, the thought process must be ‘my child is having a reaction now and I need to treat it.

I think they’re scared that they’re going to hurt the child by jamming a needle in them. Or they’re scared that they’re going to get in trouble for doing something that wasn’t necessary, right? So people’s fears need to be allayed, that you’re not going to get, you won’t be held accountable for the cost of an epi, because some people worry about things like this. Or that you’ve done something medically wrong to the child or something. Just give it. (M4)

Parents relate that one of the key features of coping with the lifestyle changes that come with living with a food allergy is learning to get over the fear of using an epinephrine auto injector. Coping in this case may mean finding ways to mitigate the costs of treatment, and exploring ways to make caregivers more comfortable with the safety of administering an auto injector.

I don’t think we realized the force you needed to push with. Which is you know, you get a trainer and it’s super loose and um but the big EpiPens you have to push a bit harder. Like the real ones. (M3)

This parent relates real world experience with using an EAI. Specifically, they relate how using an actual devise is not the same as using a trainer, and one way of coping may include becoming more comfortable by practicing with an actual device.

I think they probably do need to hear it from other parents who have been there. Cause it’s one thing to hear a nurse say it’s no problem or a doctor say just give it, but it’s, it’s also um, what else are you going to say right? But how about here’s someone who’s lived it? (M3)

## Discussion

### Global impact of anaphylaxis

Today, medicine is increasingly concentrating on the importance of quality of life as much as on health outcomes [11]. Participants described that every aspect of their life changed after their child was diagnosed with a food allergy. These findings are congruent with a previous study that identified that parents of children with food allergies found social activities outside the home more stressful [12].

Participants in this study spoke about caring for children with multiple food allergies and stated that life becomes more difficult with each food allergy. This finding echoes other studies that suggest that parents of children with multiple allergies have significantly more anxiety [13]. One participant described that it is not only food that the household needs to avoid, but also nonfood items in the home such as moisturizing creams [14].

Many parents recalled the multiple signs and symptoms of anaphylaxis and they described the helplessness they felt as they witnessed their child’s suffering. Despite having an EAI present, most expressed concern with administering a needle to their child, as they perceived that it would contribute to their child’s pain. The size of the needle was another component that was emphasized. If one assumes that the needle is the size of the cartridge, then they would certainly assume that the injection would cause significant pain and discomfort. Health care providers should describe the length of the needle found in EAIs, so that caregivers understand it is similar to the needle size used for routine immunizations. It is important for health care providers to inform caregivers that the instantaneous discomfort of a needle will quickly resolve and stop the reaction from progressing (Table 3).

**Table 3:** Barriers to epinephrine use

Emotional barriers	Health care barriers
Fear/anxiety	Limited educators (time and availability)
Guilt	Inconsistent health care information
Hesitation/doubt	Conflicting health care message
Isolation	Lack of consistent support

It is known that parental attitudes can influence their children [12]. A study on food allergy showed that maternal anxiety is associated with adolescent distress, possibly due to children learning from parental behavior [15]. Health care providers need to explain to caregivers that it is understandable to feel anxiety, but their child can learn to mimic these unhealthy behaviors. Some participants described no longer visiting the homes of people who put their child at risk of a reaction. Avoiding activities negatively reinforces anxious behaviors [16]. Unintentionally their children, adopt these negative coping skills [17]. These displays of anxious behaviors can affect a child’s relationships, school functioning and quality of life. Untreated anxiety in children can predict depression later in life [17]. It is important that health care professionals understand the psychological effects associated with living and caring for a child with food allergies, and if necessary, referrals to mental health practitioners should be supported [18].

Parents relate what they would like to see in the context of an educational tool for anaphylaxis and food allergies. They would appreciate seeing real world examples of reactions and hearing from those who have had the experience of suffering from an anaphylactic reaction or using an EAI and even hearing the subjective experience of those who have been given epinephrine to treat an anaphylactic reaction. It would also be helpful to share experiences of others who have gone through the common feelings of fear, doubt and helplessness when they have been faced with having to treat a loved one suffering a reaction. The consensus is that an educational video that includes real world experience and explores some of the common barriers that families with food allergies are faced with would be a helpful resource. Lanser conducted focus groups to assess the needs of caregivers managing food allergy and found that learning from other families was important [19] and identified the need for videos describing the experiences of others. Haigh and Hardy stated that “the importance of storytelling as the foundation of human experience cannot be overestimated” and can be used as an educational strategy [20]. This method can help other caregivers feel less isolated and prepare them for future challenges they may face. As an outcome of this study, a 3-part parent-led educational video entitled “Food Allergy: Through the eyes of a parent” was developed. This video series highlights parents’ stories of life challenges, anaphylactic reactions, and advice for other caregivers. This video is now incorporated into parent food allergy classes at the Children’s Allergy and Asthma Education Centre (http://www.caaec.ca) and is available for free on their website (https://www.youtube.com/playlist?list=PLcMGfBsVtJrAgaE5QFsg9gEhtySytDBmS).

### Health care professionals

The ways in which hospital staff interacts with caregivers impacts their experience and affects their future actions. Parents have stated that kindness, and clear communication of health care providers can positively impact an Emergency Department visit [21]. One family explained that they were yelled at for not using an EAI and this made them feel guilty and ashamed. It has been shown that negative interactions, such as insensitivity or conflict, have a greater impact on caregivers of children with a food allergy compared to positive social supports [22]. Health care providers must remember that every interaction with a patient and their family is an educational opportunity that should be utilized to teach, review and support.

This study has identified parents’ experiences of inconsistent messages from health care professionals. This inconsistency may in part be due to health care professionals treating patients without formal allergy training [23]. It is important to educate patients about the multiple symptoms of anaphylaxis and explain that any combination may occur. Past reactions will not predict future reactions and caregivers always need to be prepared to treat anaphylaxis. If health care providers teach caregivers the importance of a life-saving medication, this message and action should be consistent with the behaviors of health care providers. When caregivers see that this life saving medication is not being used by Paramedics or in the Emergency Department, this creates doubt. Why should caregivers use a needle on their child (causing pain to their loved one) when the health care providers do not use it? Having caregivers’ emotions validated by health care professionals provides ongoing support. As part of formal allergy education, there needs to be a mental health component so that caregivers understand that symptoms such as anxiety and fear are common and can be managed. This type of initiative will raise the standards of care in the management of anaphylaxis whether it is in hospitals or the community.

## Conclusions

The findings of this study indicate the need for health care professionals to address, educate and prepare parents for the myriad of emotions, thoughts and behavioral responses that are encountered when parents live with children with food allergies. When health care providers teach that these feelings are common, and reinforce the importance of prompt administration of EAIs, caregivers will be reassured that these emotions and doubts are part of the normal process of treating a child. It cannot be overstated that use of an EAI cannot hurt a child and there are no contraindications to their use. Further research into mental health and emotional communication with patient support groups and service delivery of this video or group sessions by parents or advocating parents is needed.

In a system where health care can be delivered by several different professionals, it is imperative that consistent information and treatment is given to all patients. All health care professionals must advocate and demonstrate prompt epinephrine administration. Listening to the experiences of parents who have witnessed their child go through life-threatening reactions can be an invaluable source of experience and this information can be used to help educate, support, and guide other caregivers.

## Abbreviation

EAI: epinephrine auto-injector
